# The Influence of the Side Chain Structure on the Photostability of Low Band Gap Polymers

**DOI:** 10.3390/molecules28093858

**Published:** 2023-05-03

**Authors:** Sven Bölke, Tina Keller, Florian Trilling, Michael Forster, Ullrich Scherf, Thomas Chassé, Heiko Peisert

**Affiliations:** 1 Institut für Physikalische und Theoretische Chemie, Eberhard Karls Universität Tübingen, Auf der Morgenstelle 18, 72076 Tübingen, Germany; 2 Makromolekulare Chemie (*buwMakro*) und Wuppertal Center for Smart Materials and Systems (CM@S), Bergische Universität Wuppertal, Gaussstrasse 20, 42119 Wuppertal, Germany

**Keywords:** organic solar cells, low band gap polymers, photodegradation, UV/vis, FTIR, PMIRRAS, benzodithiophene, benzothiadiazole

## Abstract

Side chains play an important role in the photo-oxidation process of low band gap (LBG) polymers. For example, it has been shown that their photostability can be increased by the introduction of aromatic-oxy-alkyl links. We studied the photostability of prototypical LBG polymers with alkyl and oxyalkyl side chains during irradiation with white light (AM 1.5 conditions) in dry air using UV/vis and IR spectroscopy. Though its degradation kinetics were distinctly affected by the presence or absence of oxygen in the structure of the side chains, in particular cases, the stability was more affected by the presence of linear or branched side chains. Moreover, we showed that the exact position of the alkyl/oxyalkyl side chain at the polymer backbone could be crucial. Although minor effects of chemical modifications on the electronic parameters (ionization potential and gap) were observed, the molecular orientation, determined by polarization modulation-infrared reflection-absorption spectroscopy (PMIRRAS), could be affected. The aggregation and crystallinity of these polymers may distinctly affect their stability.

## 1. Introduction

Low band gap (LBG) polymers have attracted huge attention for their applications in the field of polymer-based organic solar cells (OSCs) and other opto-electronic devices [1,2,3,4,5]. In this context, the photostability of conjugated polymers has been increasingly studied over the past years [1,6,7,8,9,10,11,12,13]. In view of these applications, a balance of efficiency, cost, and stability is needed [14,15].

For example, through intensive study of the photooxidation mechanism of poly-3-hexylthiophen (P3HT), two different pathways have been intensely discussed: photosensitization (most likely including the formation of singlet oxygen) and a radical chain mechanism [12,16,17,18]. The rate of this radical mechanism strongly increases towards UV light [19]. In solution, it was shown that singlet oxygen can attack the π-electron system of the P3HT backbone via cycloaddition [20,21], and superoxide radical anions were detected [22]. However, such a mechanism may depend distinctly on the detailed chemical structure of the polymer [7]. Less often, the role of side chains in the photooxidation process of polymers has been discussed [23,24,25]. For example, it has been predicted that the introduction of aromatic-oxy-alkyl links makes materials more resistant to photooxidative degradation by reducing hydrogen abstraction [26,27]. Additionally, the branching point of the side chains can play an important role, affecting the crosslinking under oxygen-free conditions [28]. Thus, side chains seem to play an important role in the understanding of the structure–property relationships impacting the photostability of conjugated materials.

**Figure 1:**
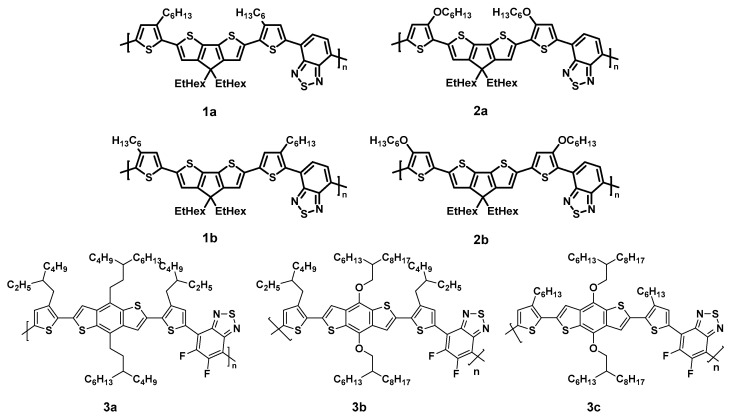
Chemical structure of the investigated LBG polymers with different side chains and positions.

In the present study, we investigate the photostability of prototypical low band gap polymers with different side chains. The studied polymers are shown in Figure 1. Due to their structural relations, they might be separated into two groups: the first group contains copolymers consisting of a backbone with cyclopentadithiophene–benzothiadiazole and thiophene moieties; its side chains are alkyl (**1a**/**1b**) or alkoxy (**2a**/**2b**) at varying positions (cf. **1a**/**2a** or **1b**/**2b**). The backbone of the second group consists of benzodithiophene (BDT) and fluorinated benzothiadiazole (BT) moieties, connected via thiophene units with branched (**3a**/**3b**) or linear (**3c**) side chains. Either branched alkyl (**3a**) or alkoxy (**3b**/**3c**) side chains are attached at the BDT moieties.

## 2. Results and Discussion

### 2.1. Electronic Structure and Molecular Orientation

Both the electronic structure, molecular ordering, and orientation of polymers may distinctly affect their stability. For example, it has been reported that a lowering of the highest occupied molecular orbital (HOMO) increases their resistance towards chemical oxidation [13]. Their molecular packing and arrangement significantly affect their photooxidative stability; [29] probably the best known example for the influence of ordering and morphology on their stability is the regioregularity of P3HT [16,30]. Moreover, these properties distinctly affect the device properties.

In this chapter, we will focus on the basic electronic parameters and orientations of polymers **3** (cf. Figure 1), and for the corresponding results for the other polymers, we refer to Ref. [31]. For the investigation into the electronic (interface) properties of a few nm thick films, two substrates with different work functions were chosen (PEI Φ = 3.3 eV and gold Φ = 5.2 ± 0.1 eV). The work function of the organic thin films on these substrates (Φ_org_), the interface dipoles Δ, and the ionization potential IP (Φ_org_ + onset of the HOMO) are summarized in Table 1. We note that, in the case of a pinning regime, Φ_org_ can be interpreted as the “integer charge transfer level” (see, e.g., Refs. [31,32] for polymers **1** and **2**). Most importantly, the structural variations in the three polymers have only a small effect on the investigated electronic (interface) properties. Additionally, the changes in the optical gap ($E_{g}^{opt})$ in Table 1, determined by ultraviolet–visible (UV/vis) spectroscopy and a Tauc plot, are rather minor. All the experimental data from the ultraviolet photoelectron spectroscopy (UPS) and UV/vis are shown in Figures S1 and S2 (Supplementary Materials). Thus, the influence of these electronic properties is most likely negligible regarding the photostability of polymers **3**. In the cases of polymers **1** and **2**, the changes are mostly related to the optical gap (1.43–1.94 eV), which will be discussed in context of the degradation behavior below.

A measure of the aggregation of the polymers in thin films might be the orientation of the molecular backbone with respect to the substrate surface. We determined the molecular orientation of polymers **3** using polarization modulation-infrared reflection-absorption spectroscopy (PMIRRAS, spectra are shown in Figure S3 in the Supplementary Materials). The data analysis was performed following the approach of Debe [33]. The method was based on the utilization of the surface selection rules of particular vibration bands near to a metal surface (e.g., Refs. [34,35,36,37]). For details on the application of this method to the polymer films, we referred to the literature [31,37]. For the analysis of the orientation vibrations at 1486, 1361 (1379 for **3a**) and 854 cm^−1^ were used. The assignment of the vibrations using DFT is summarized in Table S1 (Supplementary Materials). The angles between the polymer backbone and the sample surface were obtained from the Euler angles of the molecule’s internal cartesian coordinates, with respect to the surface normal, calculated according to Equations (S1) and (S2) (Supplementary Materials). The data in Table 2 reveal that the backbones of polymers **3** were preferably oriented parallel to the substrate surface, and the largest average tilt angle was observed for polymer **3c**. Changes in the orientation due to subsequent annealing, as is often performed to increase the degree of ordering, were almost negligible. Very similar average tilt angles (about 20°) were recently observed for polymers **1** and **2** [31]. Only for polymer **1b** was the average tilt angle significantly increased (34°) [31], which may point to a higher degree of disorder.

Comparing polymers 3 only, the orientation of the backbone was almost identical for **3a** and **3b**, and somewhat different for **3c**. This indicated a different molecular arrangement in the thin films, which may affect their photooxidation behavior. We note, however, that we have only considered the orientation of the polymer backbone. A possible influence of the conformation of the side chains will be discussed in the next section.

### 2.2. Photostability of the Investigated Low Band Gap Polymers

First, the photooxidation kinetics of all the polymer films were monitored using UV/vis absorption spectroscopy. As a consequence of the irreversible degradation of the π-conjugated system, a loss of absorbance in the UV/vis was observed. Since environmental factors (e.g., humidity and air) may have a significant influence on these degradation kinetics [10,16], all the experiments were performed in dry synthetic air under AM 1.5 conditions.

**Figure 2:**
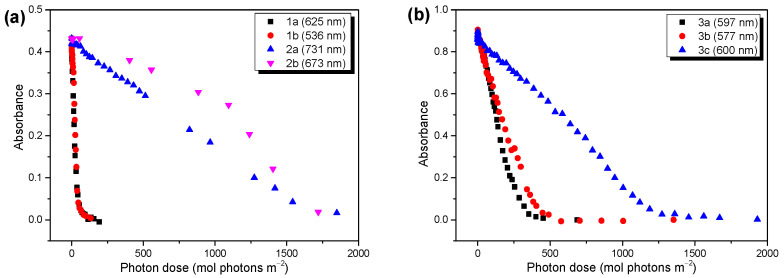
UV/vis absorbance at wavelength given in brackets as a function of the photon dose (solar simulator AM1.5). (**a**) For polymers 1 and 2, oxygen introduction in the side chain structure has a huge impact on the photostability of the LBG polymers. (**b**) Polymers 3 do not show such an effect. For those structures, linear instead of branched side chains on the thiophene units increase their photostability.

The developments of the UV/vis spectra as functions of the illumination time are summarized in Figure S4 (Supplementary Materials) for all the polymers. The UV/vis spectra of polymers 3 before degradation showed a distinct broadening with respect to the solution spectra (Figures S2 and S4, both Supplementary Materials), which can be attributed to the occurence of aggregation bands, as also observed for related (co)polymers [7,38]. The different aggregation properties of polymer **3c** might be also affected by its distinctly higher molecular weight compared to the other polymers (cf. section Materials and Methods). Therefore, the decay kinetics were not evaluated from the lowest lying features, but from the development of the absorbance maxima. The photon dose was calculated from the time trace by multiplying the intensity of the solar simulator, calibrated with a reference solar cell, by the time of irradiation. The trends of the absorbance as functions of the photon dose are summarized in Figure 2. Clearly visible, the radically different decay kinetic for polymers **1a** and **1b** compared to **2a** and **2b** are shown in Figure 2a. The differences in the average decay rates, extracted from the linear fits of the data, were huge (7.9 × 10^−3^ and 8.6 × 10^−3^ (mol photons m^−3^)^−1^ for 1a and 1b, and 2.4 × 10^−4^ and 2.1 × 10^−4^ (mol photons m^−3^)^−1^ for **2a** and **2b**). This trend followed the expected behavior very nicely, which predicted an increased photostability for the low band gap polymers with alkoxy side groups [26]. Additionally, for polymers **3a**, **3b**, and **3c**, different decay kinetics are observed in Figure 2b. However, in these cases, the introduction of oxygen to the side chain structure did not increase the photostability significantly, and polymers **3a** (alkyl) and **3b** (alkoxy) showed similar decay rates. The reaction rates, obtained from the linear fits (Figure S5, Supplementary Materials), were 2.9 × 10^−3^ and 2.1 × 10^−3^ (mol photons m^−3^)^−1^ for polymers **3a** and **3b**, respectively. In contrast, the average decay rate of polymer **3c**, which deviated from **3b** via the linear side chain at the thiophene moiety, was about three times slower compared to **3a** and **3b** (reaction rate 6.8 × 10^−4^ (mol photons m^−3^)^−1^). The reasons for the apparently surprising behavior of polymers **3** are discussed in the following sections. We also note that the electronic parameters mentioned above were not essential for the large observed differences in the degradation behavior.

**Figure 3:**
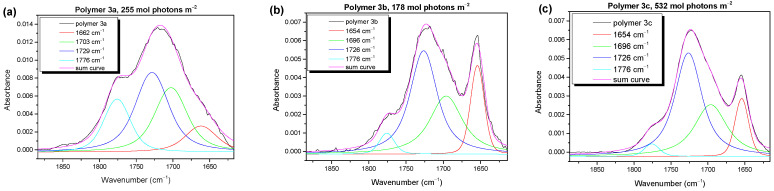
IR spectra in the carbonyl region (1900–1600 cm^−1^) described by four components, which can be ascribed to different chemical species. The ones around 1660 cm^−1^ and 1776 cm^−1^, related to main degradation product at 1728 cm^−1^, show completely different behavior for polymer **3a** (**a**) compared to **3b** (**b**) and **3c** (**c**), which indicates different degradation mechanisms due to different side chains.

Valuable information about the degradation mechanism can be gained from the infrared (IR) spectroscopy. First, we will compare the development of the (non-volatile) reaction products in the carbonyl region as a function of the irradiation time. In Figure 3, we show the typical IR spectra for the three polymers (**3a**, **3b**, **3c**) at medium degradation times.

The spectra in Figure 3 can be described by four components. For the peak fit, a Lorentzian line profile was assumed. The absorbances of these carbonyl components at the different stages of photooxidation are summarized in Figure S6 (Supplementary Materials). All the spectra exhibit main (most intense) bands at 1726–1729 cm^−1^ (blue curves) and 1696–1703 cm^−1^ (green curves). At these wavenumbers, a broad variety of different carbonyl and aldehyde species is found [19,39]. Additionally, in the region of the red component in Figure 3 (1654–1662 cm^−1^), carbonyl species were expected [19]. The component at the highest wavenumber (~1776 cm^−1^, cyan curve) might be assigned to a peracid, perester [40], or anhydride [12] species. This component was strongest for polymer **3a** (alkyl group at the BDT moiety) and less pronounced for **3b** and **3c**, with alkoxy groups at the BDT unit. This may indicate that a mechanism including an organic peroxide group was mainly present for polymer **3a** (where a formation of peroxy compounds at the BDT might be possible), whereas alternative mechanisms determined the photooxidation of polymers **3b** and **3c**. For the BDT moieties with alkyl chains (like polymer **3a**), a mechanism via peroxide formation at the aromatic ring was proposed in the literature [41]. Another striking difference in the composition of the carbonyl species for polymer **3a** and polymers **3b** and **3c** was the appearance of the component at the lowest wavenumbers (red curve in Figure 3). In former studies on P3HT photooxidation, a component at similar wavenumbers was assigned to a carbonyl species attached directly to the thiophene ring, formed after chain scission [40,42]. One might speculate that such a mechanism is especially important for the photooxidation of polymers **3b** and **3c**.

We note that the reaction products of polymers **1** and **2** also exhibited different compositions, dependent on the presence of alkyl- or alkoxy- groups (Figure S7, Supplementary Materials, for intermediate degradation times). The species at the lowest wave numbers (red) were the main features in the carbonyl spectra of polymers **1**, but were minor for polymers **2**. In this case, the different photooxidation reaction rates of polymers **1** and **2** could be related to the different degradation products and thus degradation mechanisms. In contrast, upon comparing polymers **3b** and **3c**, their different reaction rates were not associated with the different compositions of the reaction products, indicating that the mechanism did not determine the photooxidation rate in this case.

**Figure 4:**
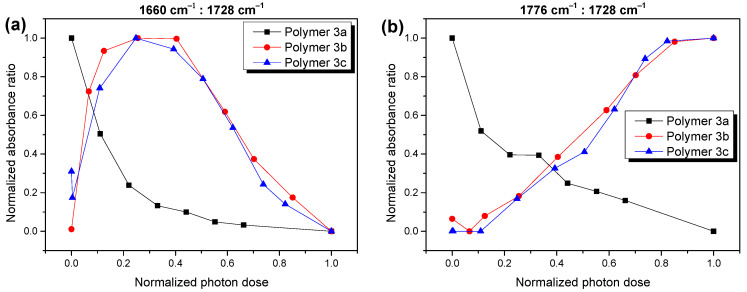
Absorbance of selected carbonyl species at 1660 cm^−1^ (**a**) and 1776 cm^−1^ (**b**) relative to the main component at 1726–1729 cm^−1^ (for better comparison values were normalized to 0 and 1). The composition of carbonyl species is significantly different for polymer **3a** compared to polymers **3b** and **3c**.

The different behavior of polymer **3a** on the one hand and **3b,c** on the other hand is even better for a visible analysis of the ratio of the different reaction products during the different stages of the photooxidation. In Figure 4, we compare the absorbance of the components at the lowest and highest wavenumbers (red and cyan curves in Figure 3), relative to the main component at 1726–1729 cm^−1^ (blue curves in Figure 3) for polymers **3a**,**b**,**c**. Although a continuous decrease in the relative absorbance for both components with an increasing degradation time was observed for polymer **3a** (black curves in Figure 4), the behavior was radically different for the cases of polymers **3b**,**c**. In the latter case, these components formed an essential part in the composition of the reaction products at the advanced stages of the photooxidation. These observations confirm that the mechanism of photooxidation was significantly different for polymer **3a** compared to **3b** and **3c**.

**Figure 5:**
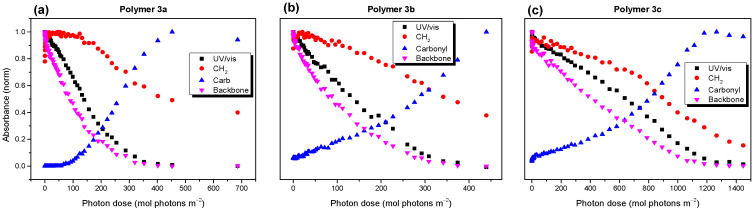
Normalized IR and UV/vis absorbance of educts and reaction products of polymers **3a** (**a**), **3b** (**b**) and **3c** (**c**) as a function of the photon dose (irradiated in dry synthetic air with a solar simulator under AM1.5 conditions). UV/vis and IR bands attributed to the polymer backbone show very similar trends.

The different photooxidation behaviors of the alkyl chains and backbones of polymers **3a** and **3b**,**c** should also be reflected in the trends of the absorbance of the specific vibrations. In Figure 5, we have evaluated the vibrational bands representing the backbones (1486 cm^−1^, cf. Table S1, Supplementary Materials) and asymmetric CH_2_ vibrations (2925 cm^-1^). In addition, the integrated absorbance in the carbonyl region and the UV/vis absorbance at the maximum was added.

The trend of the backbone-related vibration absorbance followed well the trend of the UV/vis absorbance for all three polymers. This could be expected, since an attack on the backbone could cause the destruction of the π-conjugated system, upon which the orbitals involved in the UV/vis transitions were located. The most significant difference in the behavior of the three polymer was the apparent delay of the decrease in the absorbance of the CH_2_-related vibrations with respect to the backbone. This delay was strongest for polymers **3a** and **3b** and less pronounced for polymer **3c**. Vice versa, the backbone of polymer **3c** was more stable, although the chemical structure of the backbone was the same for all three polymers.

**Figure 6:**
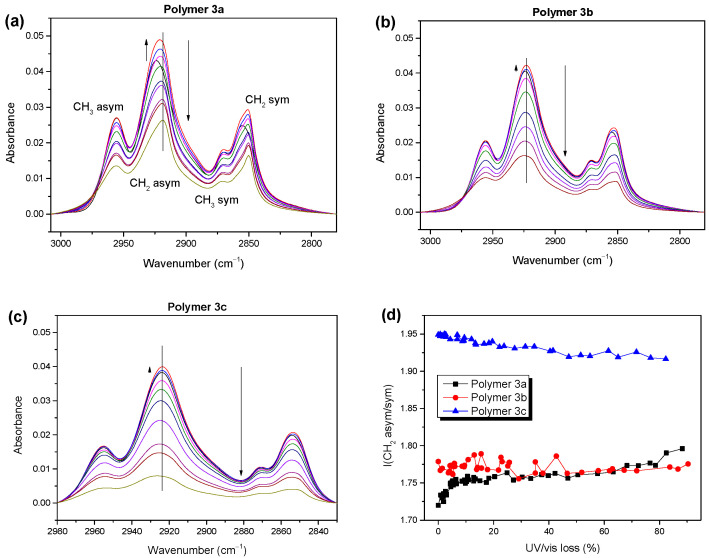
(**a**–**c**) IR spectra of polymers **3** of the alkyl region at different stages of degradation. (**d**) Ratios of IR intensities of asymmetric to symmetric CH_2_ vibrations for polymers **3** as an indicator for conformational changes in the alkyl side chains.

Surprisingly, an increase of 10–20% in the CH_2_ absorbance in the initial steps of the degradation is observed in Figure 5. A zoom into this region and the corresponding IR spectra are shown in Figure S8 (Supplementary Materials) and Figure 6a–c. Such an increase may have arisen if: (i) new compounds were formed containing C-H bonds (preferably with a high oscillator strength), or (ii) the orientation of the C-H bonds with respect to the electric field vector of the incoming IR light changed. Although significantly different intensities of the CH vibrations were observed (comparing, e.g., olefin-like species to aliphatic ones [43]), the oscillator strength of the aliphatic vibrations was rather high and the formation of additional C-H bonds seemed to be unlikely. Therefore, we are left with the scenario of a change in the orientation of the CH_2_ groups during the initial step of photooxidation. Distinct changes in the absorbance of the CH_2_ (but not CH_3_) vibrations due to conformation changes have also observed been in the literature [44]. Indeed, in our case too, the absorbance changes in the CH_3_-related vibrations were hardly visible during the initial steps of degradation (Figure 6).

The observed initial intensity increase, indicated by the small upward-pointing arrows in Figure 6, was strongest for polymer **3a** (>20% increase in the absorbance) but still significant for polymers **3b** and **3c**. For polymer **3a**, the increase in the absorbance of alkyl was accompanied by an energetic shift of about 6 cm^−1^ to lower wavenumbers (Figure 6 and Figure S9, Supplementary Materials), which may have been caused by chemical modifications. For example, a hydrogen abstraction on the alpha carbon would lead to the formation of double bonds and a shift in the C-H vibration to lower wavenumbers [42,43]. On the other hand, a change in the side chain conformation could also cause an energetic shift [44].

In addition, the detailed arrangement and coupling of the side chains could affect the intensity ratios of the asymmetric and symmetric CH_2_ vibrational bands (asym/sym) [45]. Thus, these ratios can be regarded as sensitive indicators for changes in the conformation and aggregation of a system [46,47]. The development of these ratios is summarized in Figure 6d for polymers **3a**, **3b**, and **3c**. For polymer **3a**, a clear increase during the initial steps of photooxidation was observed, pointing not only to a change in the orientation, but also to a decrease in the degree of the ordering of the side chains. Most importantly, the asym/sym ratios were generally lower for **3a** and **3b** (less than 1.8) compared to polymer **3c** (1.95). This indicates that the ordering and conformation of the side chains of polymers **3a** and **3b** were distinctly different compared to **3c**, which may explain the deviations in the molecular orientations obtained from PMIRRAS, as discussed above.

For thiophenes, a photooxidation mechanism is often proposed based on an energetically preferred attack on the side chain [12,24]. Therefore, the chemical structure of the side chain may determine the photostability of a polymer. The difference between polymer **3b** and the more stable polymer **3c** was the branched and linear side chains at their thiophene moieties, respectively. A radical might be stabilized at the tertiary carbon atom of a branched side chain, causing the lower stability of **3b** [48,49]. The mechanism might be also different for polymers with branched or linear alkyl side chains [50]. However, in the case of polymers **3**, the different orientations and aggregations, concluded from PMIRRAS and the intensity ratios of the CH_2_ bands, may significantly affect their photostability.

### 2.3. Wavelength Dependence of Degradation Mechanism of Polymer **3a**

Further information about the photooxidation mechanism can be obtained by wavelength-dependent photooxidation studies. Since the photosensitization depends directly on the number of absorbed photons, we are able to distinguish between absorbance-dependent (possibly including the formation of singlet oxygen and/or peroxides) and energy-dependent (radical chain) mechanisms. For P3HT, it has been demonstrated that the rate of its radical mechanism strongly increases towards UV light [19]. Since we obtained some hints for the formation of peroxides during the photooxidation of polymer **3a** (see above), we chose this polymer for our wavelength-dependent studies.

**Figure 7:**
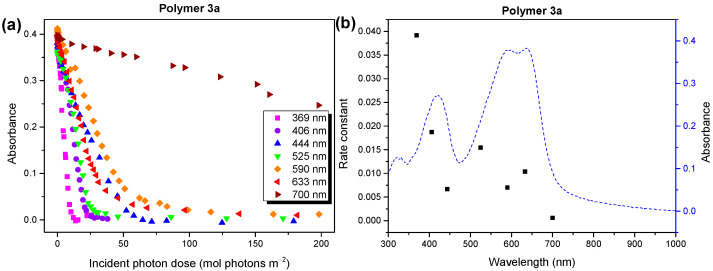
(**a**) Wavelength-dependent absorbance, and (**b**) average rate constants for the photooxidation of polymer **3a** compared to the UV/vis absorbance. Photons in the UV region lead to the fastest polymer degradation.

In Figure 7a, we show the development of the absorbance at the maxima of the UV/vis spectra, which is a function of the number of incident photons for seven different irradiation wavelengths between 369 and 700 nm. An almost linear behavior is observed; wavelength-dependent rate constants were obtained from the linear fits to the data. The rate constants are summarized in Figure 7b, together with the UV/vis absorption spectrum before degradation. We note that this data evaluation can be carried out based on the absorbed photons (Figure S10, Supplementary Materials), which leads to similar results in our case. The reaction rates in Figure 7b did not explicitly depend on the absorbance or energy of the incident photons. However, the fastest degradation rate was observed for the energy-rich UV light, indicating the domination of a radical mechanism under these conditions. Although a clear correlation of the reaction rates with the absorbance, which would indicate the occupation of long-living excited states, is hardly visible in Figure 7b, the contribution of such a photooxidation mechanism cannot be ruled out.

**Figure 8:**
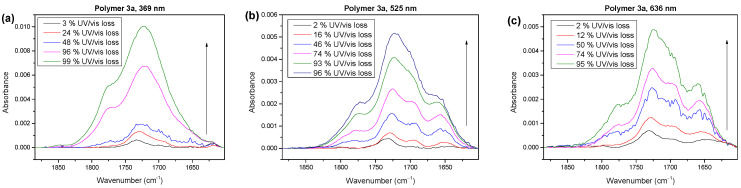
IR spectra of polymer **3a** in the carbonyl region for photooxidation at different irradiation wavelengths. (**a**) UV (369 nm) leads to a different shape, worth mentioning the very little structure around 1650 cm^−1^ compared to 525 nm (**b**) and 633 nm (**c**).

In the next step, we will compare the compositions of the reaction products in the carbonyl region for the photooxidation under UV (369 nm) and visible (525 and 633 nm) light. The corresponding spectra are shown in Figure 8 for the different stages of degradation.

The shape of the spectra in Figure 8 clearly depended on the wavelength of the irradiating light, and only minor changes in the degradation times were visible. For the irradiation with UV light (Figure 8a), the spectral shapes were very similar to the degradation under AM 1.5 conditions (cf. Figure 3a). This suggests that a radical mechanism dominated the degradation under white light. The species at around 1660 cm^−1^ for polymer **3a**, which could possibly be attributed to a peroxide, was negligible in the UV region.

In contrast, the photooxidation under visible light (Figure 8b,c) resulted in spectral shapes that were comparable with the spectra for polymers **3b** and **3c** (Figure 3b,c) under AM 1.5 conditions. The shoulder at the lowest wavenumbers was increased (red peak in Figure 3b,c), whereas the shoulder at the highest wavenumbers was decreased (cyan peak in Figure 3b,c). This indicates that, in the visible light region, the degradation mechanism of polymer **3a** was very similar to that for polymers **3b** and **3c** in white light. In other words, the mechanisms were not completely different for polymers **3a** and **3b**,**c**, but the dominating mechanism under white light conditions depended on the details of the chemical structure.

**Figure 9:**
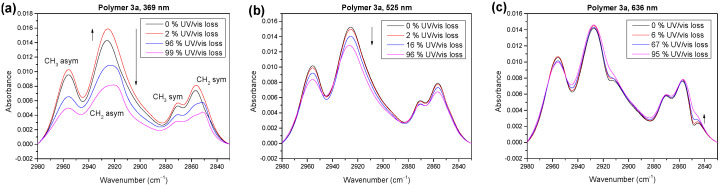
IR spectra of polymer **3a** in the alkyl region for photooxidation at different irradiation wavelengths of 369 nm (**a**), 525 nm (**b**) and 636 nm (**c**). Increase and decrease, as well as wavenumber shift, strongly depend on the wavelength.

A possible radical mechanism in the UV range might particularly attack the side chains of the polymers. In Figure 9, we compare the development of the IR spectra in the alkyl region after photooxidation at different irradiation wavelengths. Clearly visible, the absorbance of the CH_3_ and CH_2_ vibrations decreased significantly faster in the UV region compared to visible light. This indicates that the radical chain mechanism for polymer **3a** (possibly related to a H abstraction on alpha C) was strongly dependent on the wavelength and could be suppressed by a UV filter. Additionally, the shift in the wave numbers and increase in the intensity at the initial degradation steps, attributed to conformational changes, only occurred with ultraviolet light. Comparably high energy would possibly be needed to enable such effects.

## 3. Materials and Methods

### 3.1. Materials and Sample Preparation

The polymer synthesis was adopted from previously established protocols [31,51,52] and is described in detail for polymers **3b** and **3c** in the Supplementary Materials (see also Refs. [31,51,52,53,54,55,56,57]). The molecular weight distributions of the investigated polymers are summarized in Table 3.

For the photodegradation experiments with a solar simulator, the polymer thin films were doctor-blade casted in a nitrogen atmosphere with 0.5% (*w*/*w*) solutions in chloroform for polymers **1** and **2** and with 2.0% (*w*/*w*) solutions for polymers **3**. A 1.0% (*w*/*w*) solution was used for the thin films irradiated with individual wavelengths. For the films investigated with IR, CaF_2_ substrates (Korth Kristalle, Altenholz, Germany) were used. The films for the wavelength-dependent investigations at 406, 444, 590, and 700 nm were casted on a glass substrate (ThermoFisher Scientific, Waltham, MA, United States). All the substrates were cleaned using chloroform and iso-propanol and underwent a subsequent UV/ozone treatment (SEN LIGHTS Corp., Osaka, Japan, Photo Surface Processor PL16-110B-1) for 15 min before the film deposition.

The gold substrates for the UPS measurements were treated with UV/ozone for 1 h. ITO (Hoya Corporation, Tokyo, Japan, sheet resistance R = 10 Ω/□) was treated like CaF_2_, with a subsequent doctor-blade casting of polyethylenimine (PEI) with a 0.1% (*w*/*w*) solution in isobutanol at 80 °C and annealing to 110 °C for 10 min. The LBG polymer films were subsequently doctor-blade casted with a 0.2% (*w*/*w*) chloroform solution in a nitrogen atmosphere. For the orientation measurements via PMIRRAS, 0.5% (*w*/*w*) chloroform solutions and 15 min UV/ozone-treated gold substrates were used. The annealing of the films to 130 °C for 30 min was followed by a cool-down ramp of 10 °C/15 min.

### 3.2. Methods

The UPS measurements were performed in a multichamber ultra-high vacuum system (5 × 10^−10^ mbar base pressure), equipped with an Omicron hemispherical analyzer (EA 125) and a helium discharge lamp (Leybold-Heraeus, Köln, Germany, UVS10/35) using He I radiation (21.22 eV).

For the UV/vis and IR measurements, the sample was removed from the solar simulator and placed into the UV/vis spectrometer, keeping it in a dry synthetic air atmosphere. The IR measurements were performed in an evacuated chamber.

For the IR transmission and PMIRRAS measurements, a Vertex 70v spectrometer (Bruker, Billerica, MA, United States) with a PMA50 module was used. The DFT calculations for the trimers and shortened (propyl) side chains were carried out to assign experimental IR bands to the vibrational modes, using Gaussian 16 [58] at the B3LYP/6-31G* level of theory and a scaling factor of 0.97.

The UV/vis transmission measurements were performed using a Maya2000 Pro detector (Ocean Optics, Ostfildern, Germany) and a DH-2000-BAL (Mikropack, Ostfildern, Germany) light source. A DH-2000-CAL (Mikropack) light source with a cosine corrector was used for the absolute light intensity calibration in the range from 200 nm to 1050 nm.

The photooxidation experiments were carried out in custom-made degradation chambers under a continuous gas flow of dry synthetic air (Westfalen, hydrocarbon free), using a LOT LS0106 solar simulator (AM 1.5; 1000 Wm^−2^) equipped with a Xenon short-arc lamp (Osram, München, Germany, XBO).

For the calibration, a reference solar cell (ReRa Systems) was used. The wavelength-dependent degradation was performed using an array of high-power LEDs (Philips, Amsterdam, Netherlands, Luxeon Rebel).

## 4. Conclusions

We studied the influence of side chains on the photooxidation of selected low band gap polymers. As expected, the presence of alkoxy side chains instead of an alkyl side chain at a thiophene moiety increases the stability significantly. The situation is more complex for side chains at the benzodithiophene (BDT) moiety (polymers **3a**, **3b**, **3c**). Comparing polymer **3a** to polymer **3b**, the rate constants of their photooxidations are comparable, despite the different alkyl or alkoxy side chains. From the composition of the reaction products, a different dominating mechanism for the photooxidation under AM 1.5 conditions for polymer **3a** compared to **3b** and **3c** was concluded. On the other hand, the photooxidations under different wavelengths reveal that several mechanisms contribute to the degradation of the three polymers **3a**, **3b**, **3c**. However, the highest stability of polymer **3c** (alkyl group at the thiophene moiety) is most likely caused by a different aggregation in the thin films. The interplay between the molecular weights and solid-state properties (especially the photostability) of the studied copolymers is beyond the scope of this study and might be the focus of further investigations. The resulting molecular weights of the copolymers under investigation are, of course, influenced by the substitution pattern of the monomers. All the copolymers have been purified by a Soxhlet extraction before use and showed a number of average molecular weight values Mn of >10 $\frac{kg}{mol}$.

## Figures and Tables

**Table 1:** Summary of electronic parameters of polymers 3a, 3b, and 3c prepared on PEI and gold as obtained from UPS and UV/vis. All values are given in eV.

	IP			Φ_org_/Δ	
Polymer	PEI	Au	$E_{g}^{opt}$	PEI	Au
3a	5.2	5.2	2.01	3.7/+0.4	4.5/−0.7
3b	5.1	5.1	2.01	3.7/+0.4	4.4/−0.8
3c	4.9	4.9	1.90	3.7/+0.4	4.3/−0.9

**Table 2:** Average angles between the polymer backbone and the (gold) substrate surface. In all cases a preferred parallel orientation is observed.

Polymer	3a	3b	3c
room temperature	15°	14°	22°
annealed (130 °C)	15°	16°	24°

**Table 3:** Molecular weight distributions of the synthesized low band gap (LBG) polymers.

Polymer	M_n_ [kg/mol]	M_w_ [kg/mol]	M_w_/M_n_	DP
1a *	12.6	14.4	1.14	14
1b *	9.7	15.4	1.59	11
2a *	7.2	10.2	1.42	10
2b *	8.0	10.0	1.25	12
**3a ^#^**	10.3	13.4	1.30	9
**3b ^#^**	17.1	23.9	1.40	14
**3c ^#^**	16.7	58.5	3.50	14

* measured in THF; ^#^ measured in chloroform; (polymer concentration: 1.0 g/L).

## Data Availability

Additional data are available on request.

## Supplementary Materials

The following supporting information can be downloaded at: https://www.mdpi.com/article/10.3390/molecules28093858/s1, Figure S1–S15: UPS HeI spectra, UV/vis absorption spectra without degradation, PMIRRAS spectra, vibrational frequencies and assigned vibrations, PMIRRAS calculations, UV/vis spectra at different steps of degradation, linear fits of UV/vis absorbance, absorbance of carbonyl components, IR spectra of the carbonyl region, zoom into Figure 5, alkyl wavenumber shift vs. degradation, absorbed photon dose, reagents and methods [31,48,52,53,54,55,56,57].
