# Supplementary material for: The Influence of the Side Chain Structure on the Photostability of Low Band Gap Polymers
Source: Molecules. 2023 May 3;28(9):3858. doi: 10.3390/molecules28093858 (PMC10180311; doi:10.3390/molecules28093858)
Supplement: Supplementary file 1 [file molecules-28-03858-s001.zip › molecules-2331740-supplementary.pdf]

SUPPLEMENTARY MATERIALS

The Influence of the Side Chain Structure on the Photostability of Low Band Gap Polymers

Sven Bölke [1], Tina Keller [2], Florian Trilling [2], Michael Forster [2], Ullrich Scherf [2], Thomas Chassé [1] and Heiko Peisert [1,*]

[1] Institut für Physikalische und Theoretische Chemie, Eberhard Karls Universität Tübingen, Auf der Morgenstelle 18, 72076 Tübingen, Germany

[2] Makromolekulare Chemie (*buwMakro*) und Wuppertal Center for Smart Materials and Systems (CM@S), Bergische Universität Wuppertal, Gaussstrasse 20, 42119 Wuppertal, Germany

* Correspondence: heiko.peisert@uni-tuebingen.de

Content

• UPS HeI spectra

• UV/vis absorption spectra without degradation

• PMIRRAS spectra

• vibrational frequencies and assigned vibrations

• PMIRRAS calculations

• UV/vis spectra at different steps of degradation

• linear fits of UV/vis absorbance

• absorbance of carbonyl components

• IR spectra of the carbonyl region

• zoom into Figure 5

• alkyl wavenumber shift vs. degradation

• absorbed photon dose

• reagents and methods.

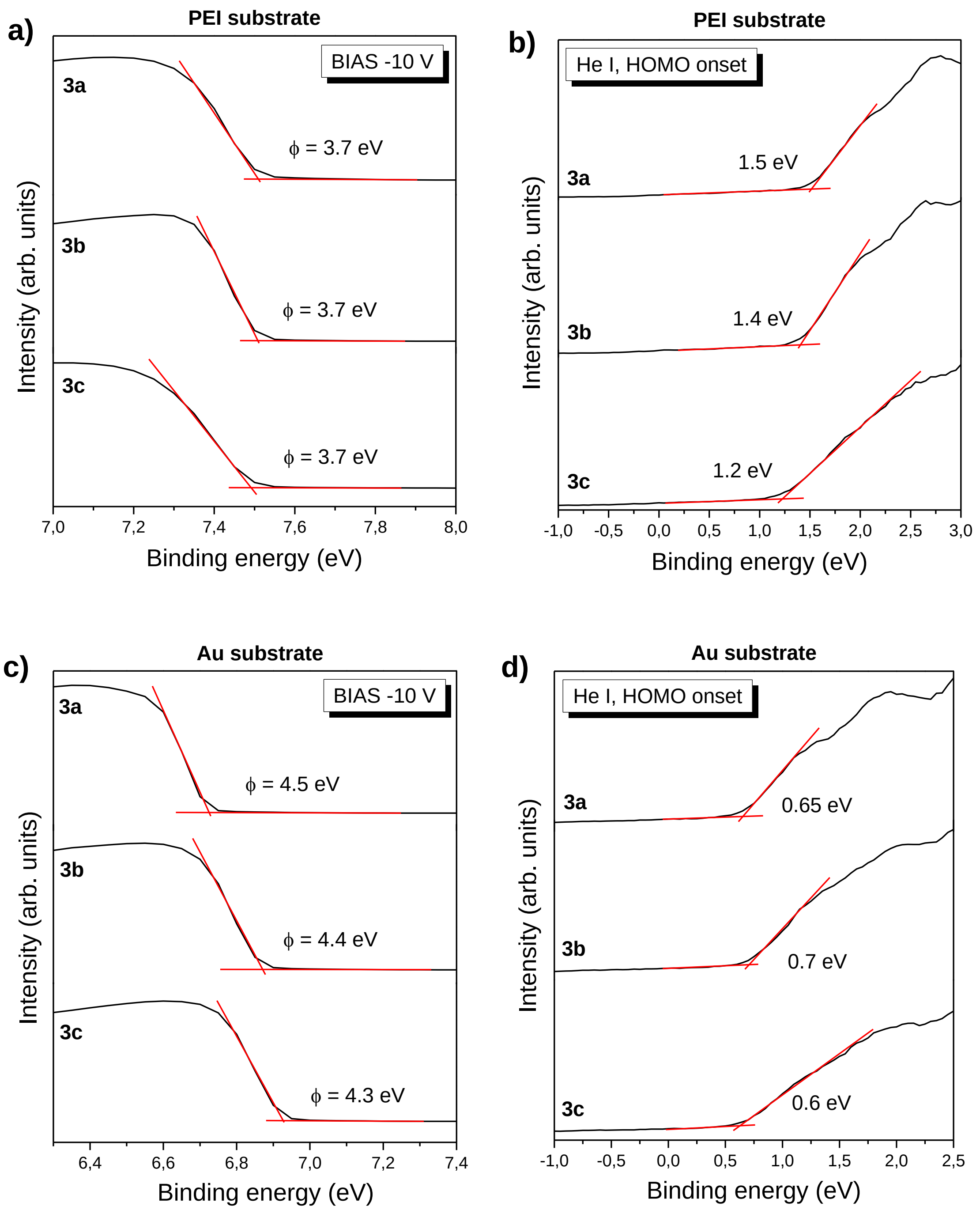

Figure S1. *UPS He I spectra for determination of work function and HOMO onset on Au (Φ = 5.2 ± 0.1 eV) and PEI (Φ = 3.3 eV) substrate.*

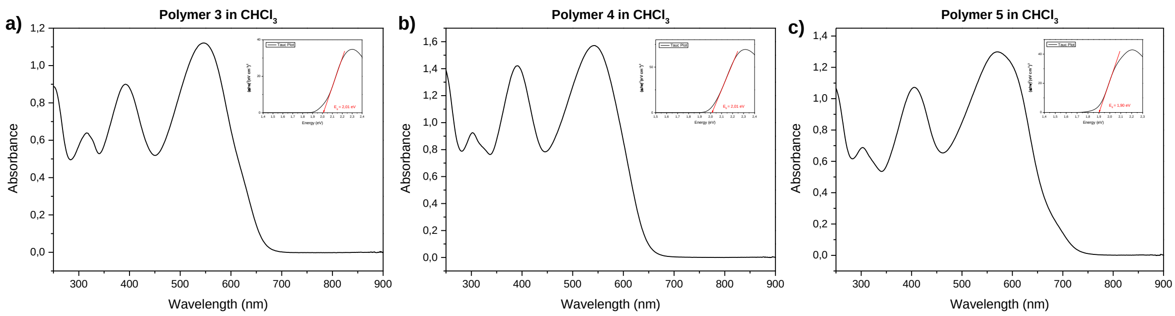

Figure S2. *UV/vis spectra of polymers in chloroform and Tauc plot for determination of* E_g^{opt} *as inset.*

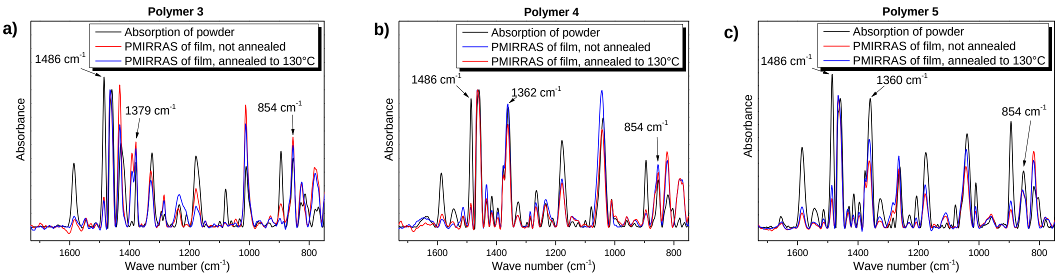

Figure S3*. PMIRRAS spectra of KBr pellets and (annealed) thin films with wavenumbers of bands used for orientation determination.*

Table S1. *Vibrational frequencies (cm^{-1}) and their assigned vibrations obtained from DFT calculations.*

3a	3b	3c	assignments
780	780	779	N=S=N/C-C/C=C/C-S str
814	804	804	N=S=N/C-C/C=C/C-F str sym (BT)
-	820	818	N=S=N/C-C/C=C/C-F str sym (BT), C-H bending ip (T)
827	-	-	N=S=N/C-C/C=C/C-S str, C-H bending oop (T/BDT)
854	854	854	N=S=N/C-C/C=C str as (BT), C-H bending oop (T/BDT)
894	894	894	C-C/C=C/C-S str as (T/BDT), CH_2 rocking
1012	1012	1011	N=S=N/C-C/C=C/C-F str sym (BT)
-	1041	1041	C-C/C=C/C-O str (BDT)

1060	1060 (shoulder)	1060 (shoulder)	CH bending ip, C-C/C=C/C-F/C-S str (T/BT)
1081	1080	1079	CH_2 twisting, C-C/C=C str (T)
1178	1182	1178	C-H bending ip (T/BDT), C-C/C=C/C-F/C-S str (T/BT)
1211	1212	1207	C-H bending ip/str (T/BDT)
1236	1234	1231	CH_2 twisting, C-H bending ip
1285	1285	1285	CH_2 wagging, C-C/C=C str (BT)
1324	1326	1328	C-C/C=C str (T/BT/BDT), C=N str (BT)
1362	1362	1360	CH_2 wagging, C-C/C=C str (BDT)
1379	1377	1378	C-C/C=C str (T/BT)
1393	1395	1395	CH_3 umbrella
1419	1416	1416	C-C/C=C str (T/BT/BDT), C-H bending ip (T/BDT)
1432	1433	1434	C-C/C=C str (T/BT/BDT); CH_2 twisting/wagging (only polymer 3b and 3c)
1459	1460	1458	C-C/C=C str (T/BT/BDT)
1486	1486	1486	C=N str as (BT), C-C/C=C str (T/BDT)
1542	1544	1540	C-C/C=C str as (T/BT/BDT), C-H bending ip (T)
1585	1586	1585	C-C/C=C str as (T/BT)

For determination of the molecular orientation with PMIRRAS, the Euler angles Ψ (angle of x rotated around the surface normal) and Θ (angle between z and the surface normal) of the

molecule's internal cartesian coordinates with respect to the surface normal were calculated according to equations (1) and (2) with the intensity ratio r:

$$sin^2\Psi = \frac{1}{1+r(zx)} \quad (1)$$

$$sin^2\Theta = \frac{1+r(zx)}{1+r(yx)+r(zx)} \quad (2)$$

x is the intensity of the IR signal at 1486 cm^{-1}, y at 854 cm^{-1} and z at 1361 cm^{-1} (1379 cm^{-1} for 3a).

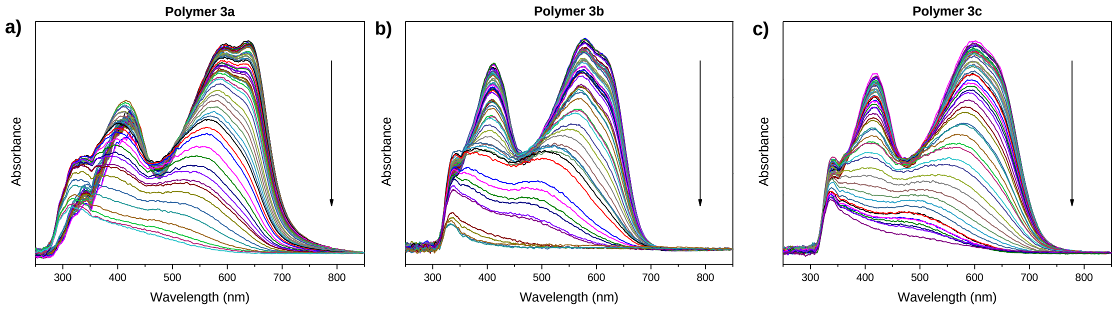

Figure S4. *UV/vis spectra of polymer group 3 without degradation (spectrum with highest absorbance, respectively) and at different steps of degradation, whereas the arrow indicates the progress of degradation(time).*

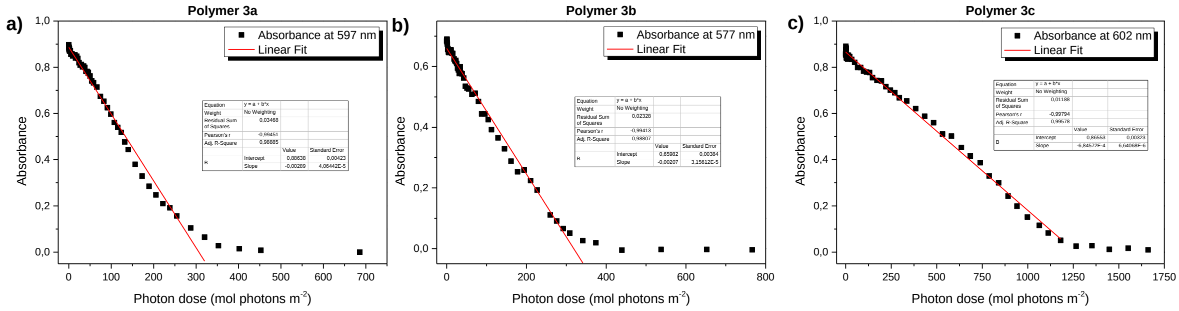

Figure S5. *Linear fits of UV/vis absorbance intensities decreasing while irradiation with white light.*

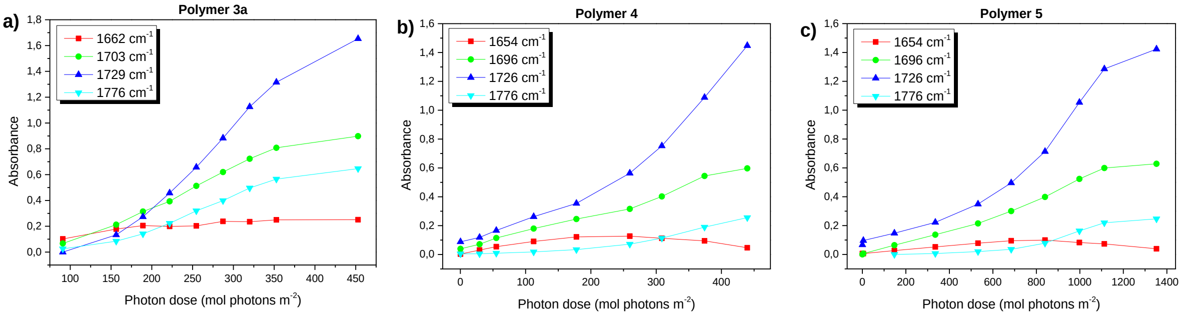

Figure S6. *Absorbance of carbonyl components as fitted in Figure 3 of the manuscript at different stages of photooxidation.*

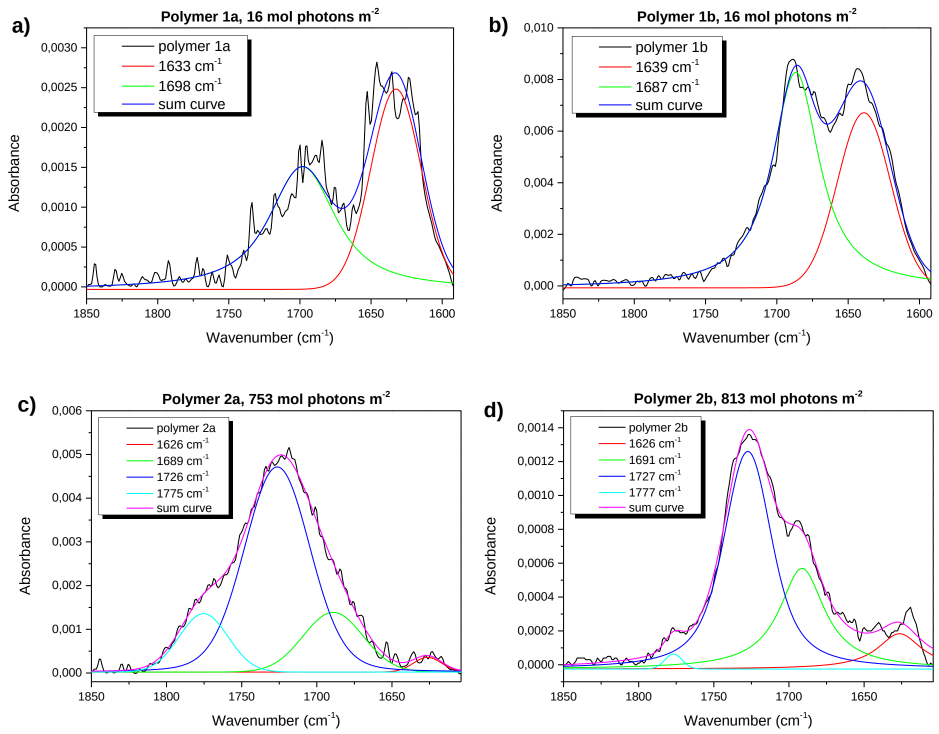

Figure S7. *IR spectra of the carbonyl region of polymers 1 and 2 at medium degradation times.*

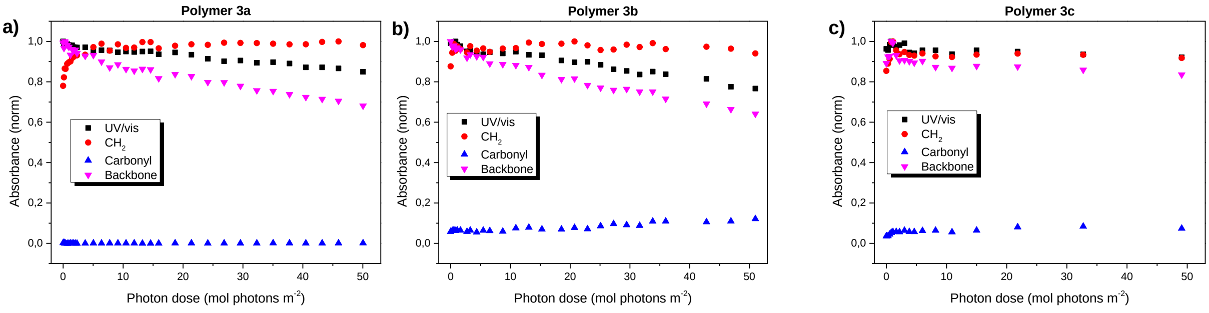

Figure S8. *Zoom into Figure 5.*

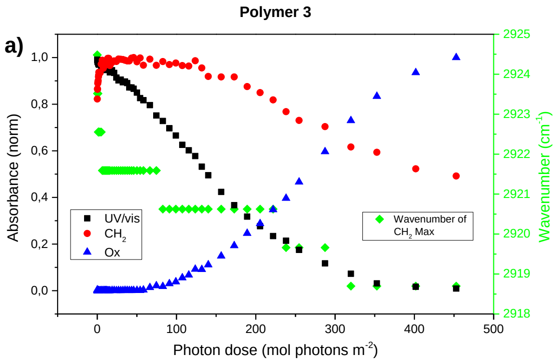

Figure S9. *Correlation of alkyl wavenumber shift and degradation/conformation change for polymer 3a.*

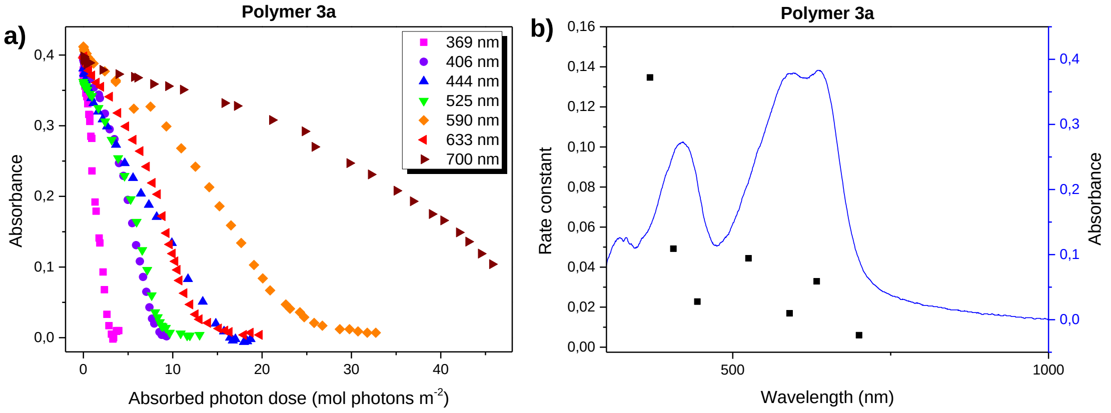

Figure S10. *a) Wavelength dependent absorbance applied against absorbed photon dose calculated after equation 1 and b) rate constants obtained from linear fits of a) compared to the UV/vis absorbance.*

$$N_{abs} = \int_{\lambda_1}^{\lambda_2} N_{inc}(\lambda) \cdot (1 - 10^{-A_\lambda})\, d\lambda \text{ (eq. 1)}$$

- N_{abs} = amount of absorbed photons
- N_{inc} = amount of incident photons
- A_λ =Absorbance at wavelength λ

Synthesis of the studied polymers

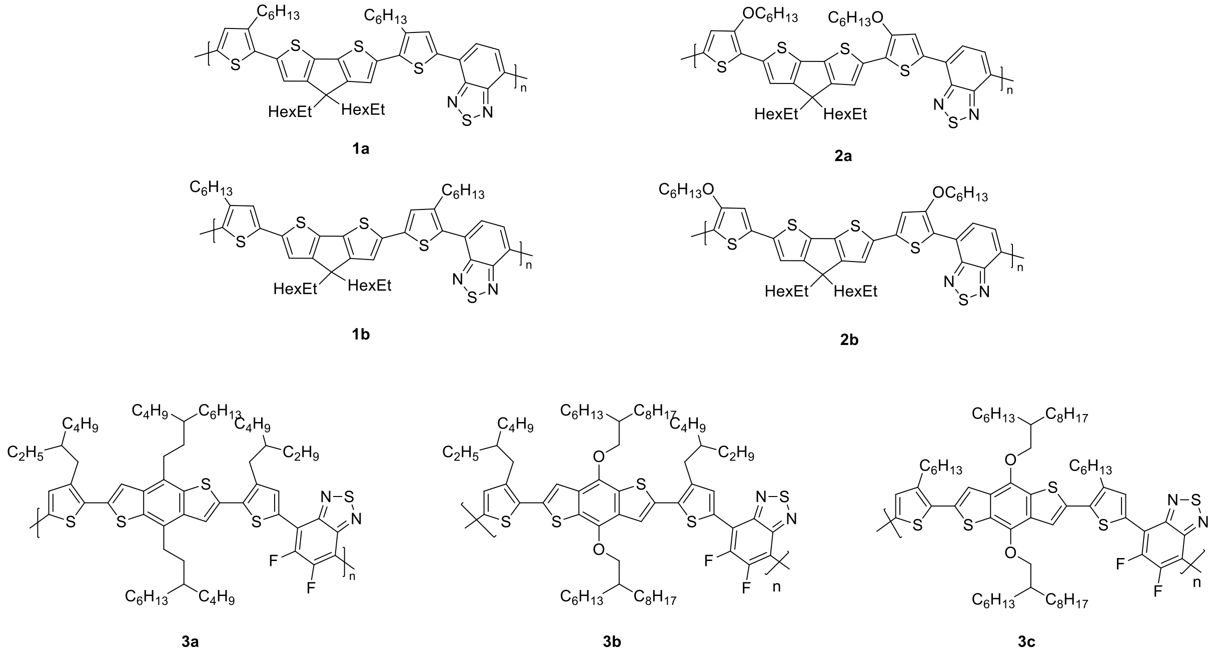

Figure S11. *Overview of the synthesized low bandgap (LBG) polymers.*

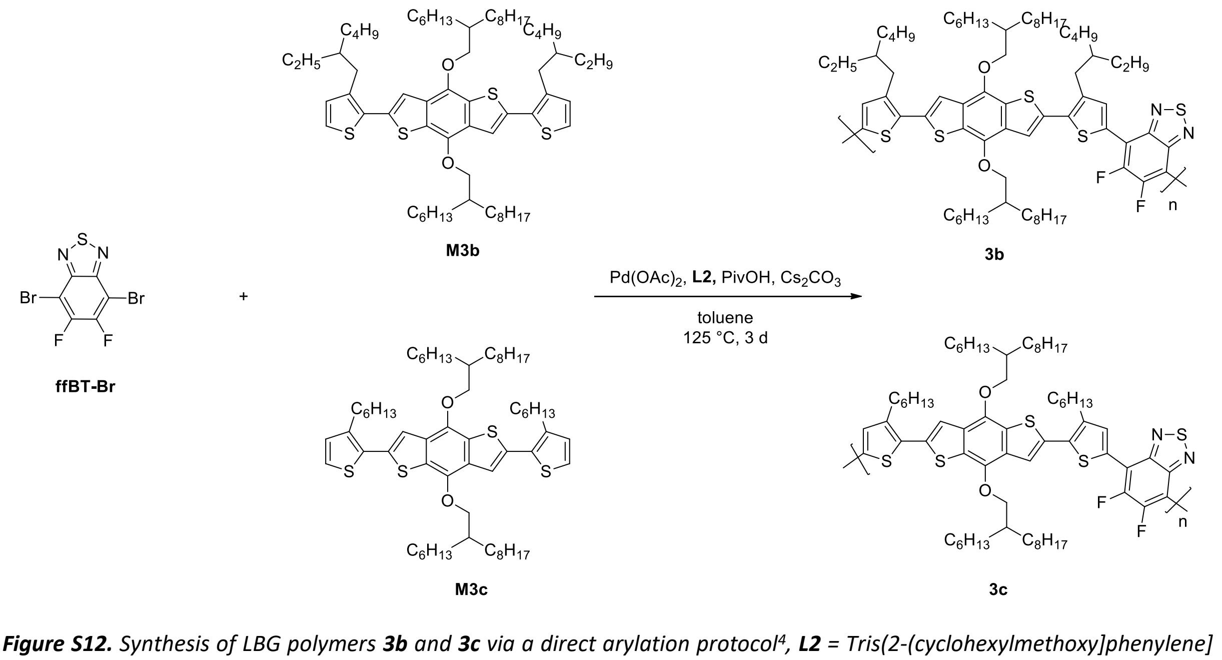

Figure S12. *Synthesis of LBG polymers* ***3b*** *and* ***3c*** *via a direct arylation protocol[4],* ***L2*** *= Tris(2-(cyclohexylmethoxy]phenylene] phosphine.*

Reagents and Methods

The polymers **1a**, **1b**, **2a**, **2b**, **3a**[31,48,52] as well as 4,7-dibromo-5,6-difluoro-2,1,3-benzothiadiazole[54], and tris[2-(cyclohexylmethoxy)phenyl]phosphine[53] were synthesized according to literature procedures or modifications of them.

The polymers **3b** and **3c** were generated in a direct arylation polycondensation (DAP) with 4,7-dibromo-5,6-difluoro-2,1,3-benzothiadiazole and 4,8-bis[(2-hexyldecyl)-oxy]-2,6-bis-[3-(2-ethylhexyl-thiophen-2-yl)]-benzo-[1,2-b:4,5-b′]dithiophene (M3b), or 4,8-bis[(2-hexyl-decyl)oxy]-2,6-bis-[3-(hexylthiophen-2-yl)]-benzo-[1,2-*b*:4,5-*b′*]dithiophene (M3c), respectively.[55,56,57]

2-Bromo-3-hexylthiophene, 2-bromo-3-(2-ethylhexyl)thiophene, palladium acetate, tetrakis-(triphenylphosphine) palladium(0), pivalic acid, and cesium carbonate and all solvents were purchased from TCI Europe Research Chemicals, Thermo Fisher Scientific and used as received. The reactions were carried out under an argon atmosphere by use of standard Schlenk techniques. The NMR spectra were recorded on Bruker Avance 400 or Avance III 600 spectrometers. Molecular weights were determined by gel permeation chromatographic analysis (GPC) with a PSS/SECcurity GPC System utilized PS-columns (two columns, 5 µm gel, pore widths mixed bed linear) connected with UV/Vis and RI detection. All GPC analyses were performed on solutions of the polymers in THF or chloroform at 30 °C (concentration of the polymer: approx. 1.0 g/L). The calibration was based on polystyrene standards with narrow molecular weight distribution. The HOMO energy levels of the polymers were determined by photoelectron spectroscopy under atmospheric conditions using a Riken Keiki photoelectron spectrometer (AC-2).

Synthesis of 4,8-Bis[(2-hexyldecyl)oxy]-2,6-bis-[3-(2-ethylhexylthiophene-2-yl)]-benzo[1,2-b:4,5-b]-dithiophene (M3b)

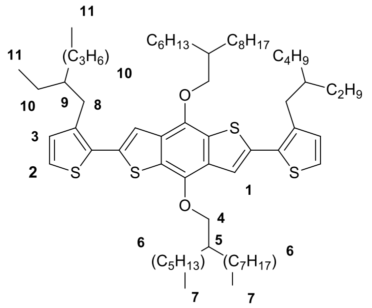

Under inert gas atmosphere and exclusion of light 2,6-bis(trimethylstannyl)-4,8-bis[(2-hexyldecyl)oxy]-benzo[1,2-b:4,5-b']dithiophene (2.0 g, 2.0 mmol, 1.0 eq.) was dissolved in 25 mL of dry toluene, and 2-bromo-3-(2-ethylhexyl)thiophene (1.41 g, 5.0 mmol, 2.5 eq.) was added. Tetrakis(triphenyl-phosphine)palladium(0) (0.19 g, 0.2 mmol, 0.04 eq.) was dissolved in 10 mL of dry toluene under exclusion of light and added to the reaction mixture. The reaction mixture was heated under reflux at 135 °C for 12 h. Subsequently, the solvent was removed. After purification by column chromatography over silica gel using n-hexane as eluent, the product was obtained as a yellow oil in a yield of 42% (0.89 g, 0.8 mmol).

^{1}H-NMR (600 MHz, $C_2D_2Cl_4$, 300 K): δ [ppm] = 7.43 (s, 2H, H_1), 7.28 (d, J = 5.2 Hz, 2H,H_3), 6.98 (d, J = 5.2 Hz, 2H, H_2), 4.19 (d, J = 6.6 Hz, 4H, H_4), 2.63 (d, J = 7.1 Hz, 4H, H_8), 1.77-1.71 (m, 4H, $H_{5/9}$), 1.57-1.25 (m, 68H, $H_{6/10}$), 0.92-0.84 (m, 24H, $H_{7/11}$). ^{13}C{^{1}H}-NMR (151 MHz, $C_2D_2Cl_4$, 300 K): δ [ppm] = 144.1, 140.1, 136.3, 132.0, 131.4, 131.2, 130.1, 125.1, 119.0, 76.7, 40.7, 39.5, 32.2, 31.6, 30.4, 30.1, 30.0, 29.7, 29.0, 27.3, 27.3, 23.4, 23.0, 14.5, 14.5, 11.2. MS (APCI): m/z $[M+H]^+$ = 953.6444 (calc. m/z $[M+H]^+$ = 953.6427) [$C_{58}H_{96}O_2S_4$+H].

Synthesis of 4,8-Bis[(2-hexyldecyl)oxy]-2,6-bis-(3-hexylthiophene-2-yl)-benzo[1,2-b:4,5-b]-dithiophene (M3c)

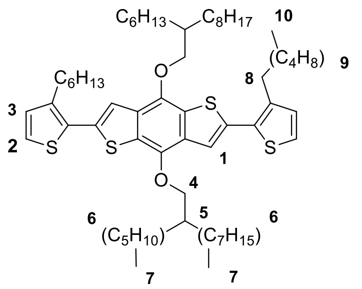

Under inert gas atmosphere and exclusion of light, 2,6-bis(trimethylstannyl)-4,8-bis[(2-hexyldecyl)oxy]-benzo[1,2-b:4,5-b']dithiophene (0.99 g, 1.0 mmol, 1.0 eq.) and 2-bromo-3-hexylthiophene (0.61 g, 2.5 mmol, 2.5 eq.) were dissolved in 12 mL of dry toluene. Tetrakis(triphenyl-phosphine)palladium(0) (0.09 g, 0.1 mmol, 0.1 eq.) was dissolved in 5 mL of dry toluene under exclusion of light and added. The reaction mixture was stirred for 12 h at 135 °C. After cooling to room temperature, the solvent was removed. Column chromatographic purification was carried out over silica gel with n-hexane as eluent. The product was obtained as a yellow oil in a yield of 40% (0.40 g, 0.4 mmol).

^{1}H-NMR (600 MHz, $C_2D_2Cl_4$, 300 K): δ [ppm] = 7.44 (s, 2H, H_1), 7.28 (d, J = 4.2 Hz, 2H,H_3), 7.01 (d, J = 5.2 Hz, 2H, H_2), 4.20 (d, J = 5.2 Hz, 4H, H_4), 2.91-2.87 (m, 4H, H_8),1.86-1.85 (m, 2H, H_5), 1.69-1.27 (m, 64H, $H_{6/9}$), 0.89-0.86 (m, 18H, $H_{7/10}$). ^{13}C{^{1}H}-NMR (151 MHz, $C_2D_2Cl_4$, 300 K): δ [ppm] = 144.0, 143.8, 141.3, 133.0, 132.1,130.8, 130.8, 124.8, 118.5, 76.5, 39.4, 32.2, 32.0, 31.7, 30.0, 29.7, 29.6, 27.3, 23.1, 23.0, 23.0,14.5, 14.5. MS (APCI): m/z $[M+H]^+$ = 1003.6535 (calc. m/z $[M+H]^+$ = 1003.6522) [$C_{62}H_{98}O_2S_4$+H].

Synthesis of polymer 3b

In a flame-dried microwave vessel with stirring bar and septum 4,8-bis[(2-hexyldecyl)oxy]-2,6-bis[3-(2-ethylhexylthien-2-yl)]-benzo[1,2-*b*:4,5-*b'*]dithiophene (400.0 mg, 376 µmol, 1.0 eq.), 4,7-dibrom-5,6-difluor-2,1,3-benzothiadiazole (124.0 mg,3 76 µmol, 1.0 eq.), tris[2-(cyclohexylmethoxy)-phenyl]phosphine (45.0 mg, 75 µmol, 0.2 eq.), palladium acetate (4.2 mg, 19 µmol, 0.05 eq.), pivalic acid (38.4 mg, 376 µmol, 1.0 eq.) and cesium carbonate (358.4 mg, 1.1 mmol, 3.0 eq.) were dissolved in 3,5 ml of dry toluene and stirred for 72 h at 125 °C. After cooling to room temperature, the solvent was removed. Then it was taken up in a few ml chloroform. The crude polymer was precipitated into cold methanol and then filtered off. Via Soxhlet extraction, the polymer was fractionated successively with methanol, acetone, ethyl acetate, n-hexane, dichloromethane, chloroform and chlorobenzene. The polymer was obtained in the form of a purple colored solid in a yield of 79 % **3b** (364.7 mg, 296 µmol, in the *n*-hexane fraction).

^{1}H-NMR (600 MHz, $C_2D_2Cl_4$, 353 K): δ [ppm] = 8.24-8.18 (m, 2H), 7.68-7.62 (m, 2H),4.34-4.16 (m, 4H), 2.95-2.85 (m, 4H), 1.93-1.24 (m, 72 H), 0.94-0.82 (m, 24H) [Homocoupling defect signals: BT-BT: 8.8, 8.3 ppm, BDT-DT: 7.8-6.7 ppm]. ^{13}C{^{1}H}-NMR (151 MHz, $C_2D_2Cl_4$, 353 K): δ [ppm] = 144.5, 140.9, 138.5, 138.1, 137.1,135.6, 132.2, 130.8, 130.2, 128.9, 126.6, 119.9, 77.5, 40.8, 39.8, 34.3, 33.2, 32.1, 31.9, 30.3,30.1, 29.8, 29.4, 29.1, 27.7, 27.3, 26.8, 23.3, 23.0, 22.8, 14.2.GPC (chloroform): M_n [g/mol] = 17,100, M_w [g/mol] = 23,900, PDI = 1.4. UV/Vis (o-DCB): $\lambda_{max.abs}$ [nm] = 393, 541. PL (o-DCB) $\lambda_{max.em.}$ [nm] ($\lambda_{exc.}$ = 560 nm) = 698. UV/Vis (film) $\lambda_{max.abs.}$ [nm] = 418, 587, 623. PL: ($\lambda_{exc.}$ = 560 nm): $\lambda_{max.em.}$ [nm] = 705 (film). PESA (AC2): E_{HOMO}: = -5.11 eV; E_{LUMO} = -3.0 eV.

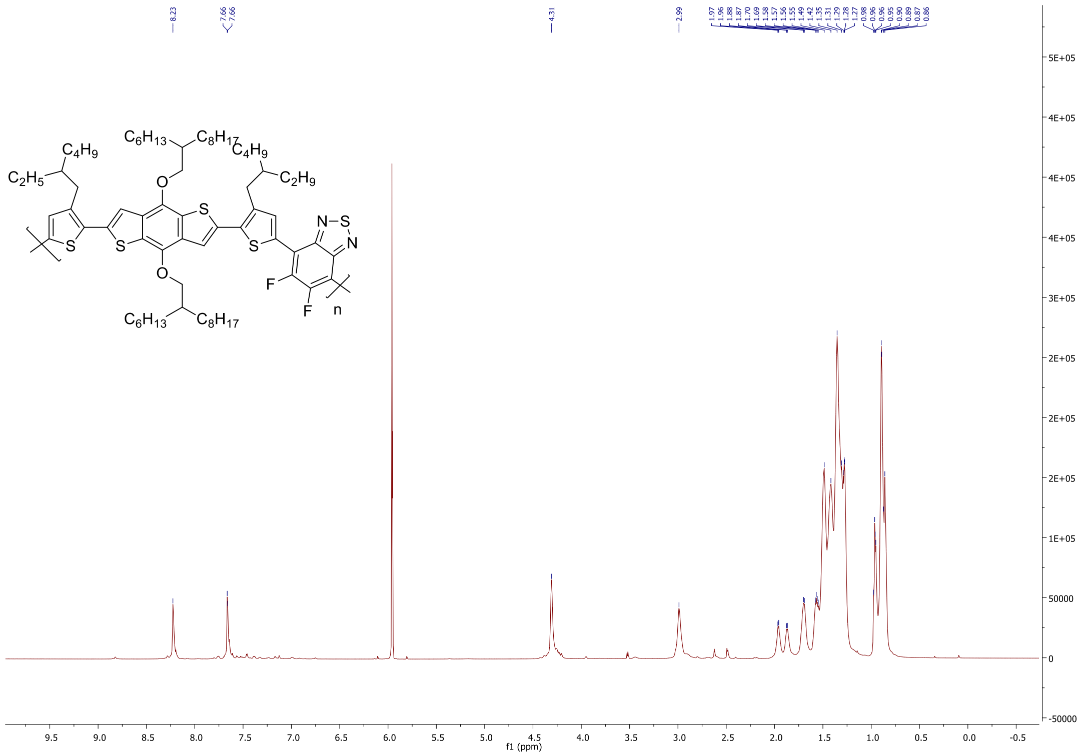

Figure S13. *^{1}H-NMR spectrum of polymer **3b** measured in $C_2D_2Cl_4$ (600MHz).*

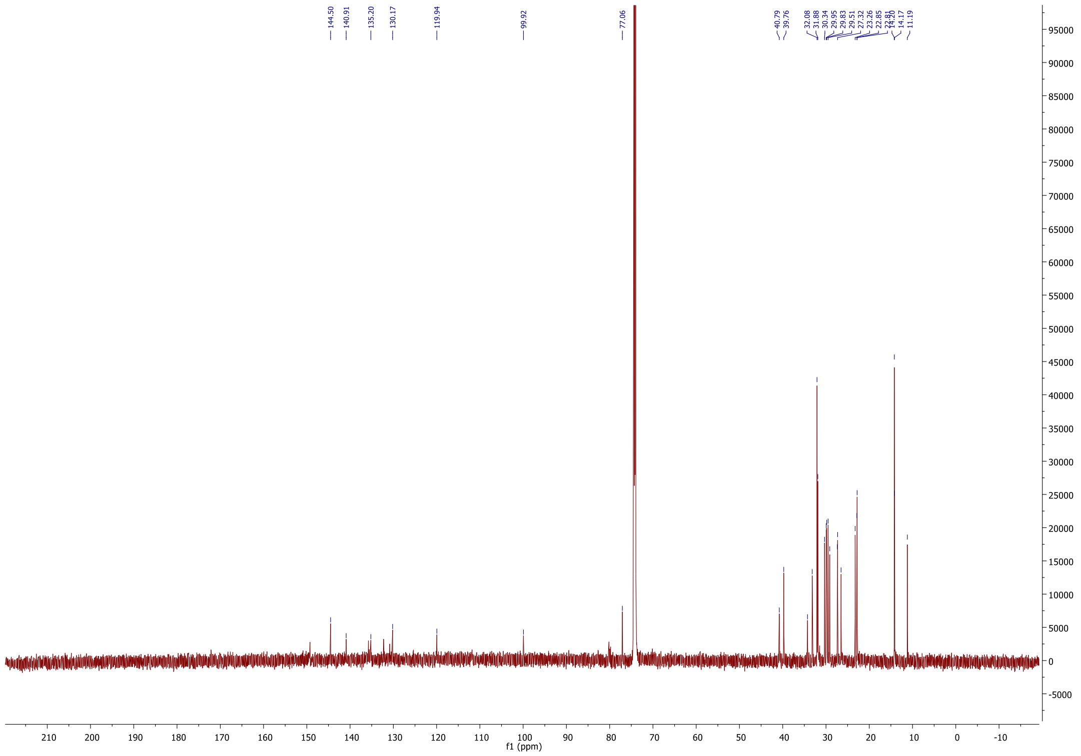

Figure S14. *^{13}C-NMR spectrum of polymer 3b measured in $C_2D_2Cl_4$ (151MHz).*

Synthesis of polymer 3c

In a flame-dried microwave vessel with stirring bar and septum 4,8-bis[(2-hexyldecyl)oxy]-2,6-bis(3-hexylthien-2-yl)]-benzo[1,2-*b*:4,5-*b*']dithiophene (389.9 mg, 388 µmol, 1.0 eq.), 4,7-dibrom-5,6-

difluor-2,1,3-benzothiadiazole (128.0 mg, 388 µmol, 1.0 eq.), tris[2-(cyclohexyl-methoxy)phenyl]-phosphine (46.5 mg, 78 µmol, 0.2 eq.), palladium acetate (4.4 mg, 19 µmol, 0.05 eq.), pivalic acid (39.6 mg, 388 µmol, 1.0 eq.) and cesium carbonate (391.0 mg, 1.2 mmol, 3.0 eq.) were dissolved in 4 ml of dry toluene and stirred for 72 h at 125 °C. After cooling to room temperature, the solvent was removed. Then it was taken up in a few ml chloroform. The crude polymer was precipitated in to cold methanol and then filtered off. By Soxhlet extraction, the polymer was fractionated successively with methanol, acetone, ethyl acetate, n-hexane, dichloromethane, chloroform and chlorobenzene. The polymer was obtained in the form of a purple colored solid in a yield of 49 % for **3c** (222.1 mg, 189 µmol, in the chloroform fraction).

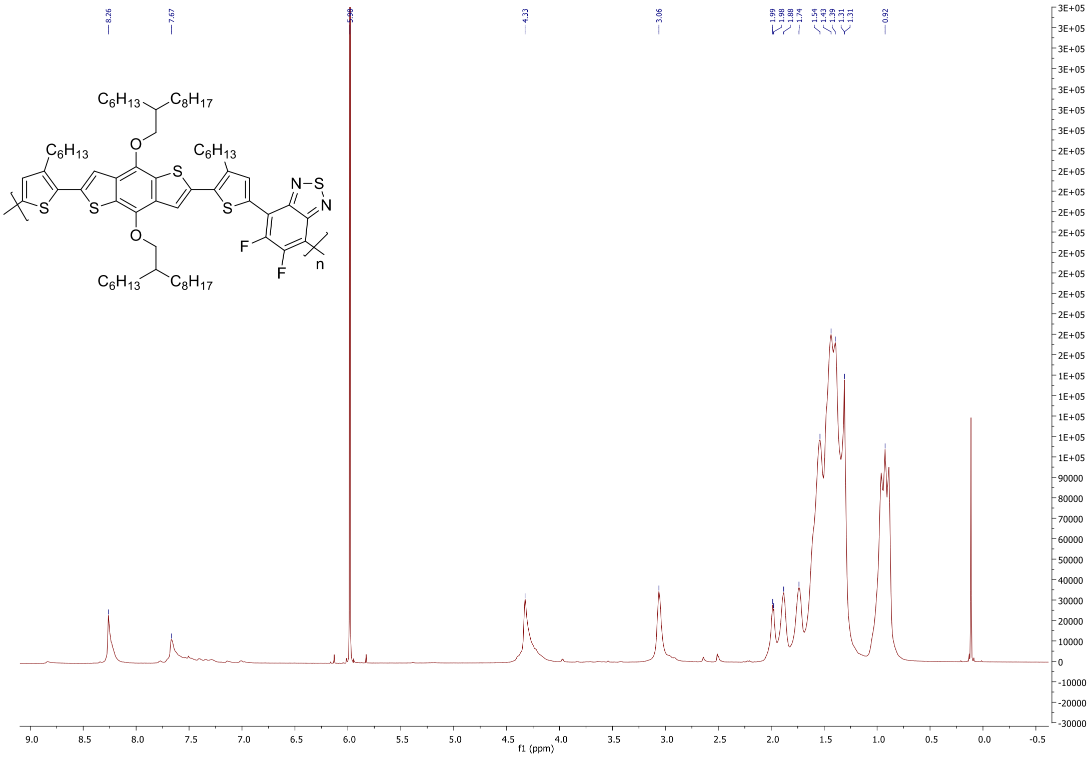

Figure S15. *^{1}H-NMR spectrum of polymer **3c** measured in $C_2D_2Cl_4$ (600MHz). (a ^{13}C-NMR spectrum of suitable could not be recorded due to solubility limitations).*

^{1}H-NMR (600 MHz, $C_2D_2Cl_4$, 353 K): δ [ppm] = 8.30-8.10 (m, 2H), 7.71-7.53 (m, 2H),4.37-4.15 (m, 4H), 3.04-2.86 (m, 4H), 1.96-1.29 (m, 66H), 0.94-0.87 (m, 18H) [Homocoupling defect signals: BT-BT: 8.82, 8.32 ppm, BDT-DT: 7.51-6.90 ppm]. GPC (chloroform): M_n [g/mol] = 16,700, M_w [g/mol] = 58,500, PDI = 3.5. UV/Vis (o-DCB): $\lambda_{max.abs}$ [nm] = 405, 562. PL (o-DCB) $\lambda_{max.em.}$ [nm] ($\lambda_{exc.}$ = 380 nm) = 706. UV/Vis (film) $\lambda_{max.abs.}$ [nm] = 423, 607, 643. **PL**: ($\lambda_{exc.}$ = 580 nm): $\lambda_{max.em.}$ [nm] = 737 (film). PESA (AC2): E_{HOMO}: = - 4.95 eV; E_{LUMO} = -2.95 eV.

References (numbers refer to the main manuscript):

31. Bölke, S.; Batchelor, D.; Früh, A.; Lassalle-Kaiser, B.; Keller, T.; Trilling, F.; Forster, M.; Scherf, U.; Chassé, T.; Peisert, H., Influence of the Side Chain Structure on the Electronic Structure and Self-Organization Properties of Low Band Gap Polymers. *ACS Applied Energy Materials* **2022,** 5, (12), 15290-15301.

48. Zhou, H. X.; Yang, L. Q.; Stuart, A. C.; Price, S. C.; Liu, S. B.; You, W., Development of Fluorinated Benzothiadiazole as a Structural Unit for a Polymer Solar Cell of 7% Efficiency. *Angewandte Chemie-International Edition* **2011,** 50, (13), 2995-2998.

52. Carlé, J. E.; Helgesen, M.; Zawacka, N. K.; Madsen, M. V.; Bundgaard, E.; Krebs, F. C., A comparative study of fluorine substituents for enhanced stability of flexible and ITO-free high-performance polymer solar cells. *Journal of Polymer Science Part B: Polymer Physics* **2014,** 52, (13), 893-899.
53. Bura, T.; Beaupre, S.; Legare, M. A.; Quinn, J.; Rochette, E.; Blaskovits, J. T.; Fontaine, F. G.; Pron, A.; Li, Y. N.; Leclerc, M., Direct heteroarylation polymerization: guidelines for defect-free conjugated polymers. *Chemical Science* **2017,** 8, (5), 3913-3925.
54. Kim, J.; Yun, M. H.; Kim, G. H.; Lee, J.; Lee, S. M.; Ko, S. J.; Kim, Y.; Dutta, G. K.; Moon, M.; Park, S. Y.; Kim, D. S.; Kim, J. Y.; Yang, C., Synthesis of PCDTBT-Based Fluorinated Polymers for High Open-Circuit Voltage in Organic Photovoltaics: Towards an Understanding of Relationships between Polymer Energy Levels Engineering and Ideal Morphology Control. *ACS Appl. Mater. Interfaces* **2014,** 6, (10), 7523-7534.
55. Wang, Q. F.; Takita, R.; Kikuzaki, Y.; Ozawa, F., Palladium-Catalyzed Dehydrohalogenative Polycondensation of 2-Bromo-3-hexylthiophene: An Efficient Approach to Head-to-Tail Poly(3-hexylthiophene). *J. Am. Chem. Soc.* **2010,** 132, (33), 11420-11421.
56. Campeau, L. C.; Parisien, M.; Leblanc, M.; Fagnou, K., Biaryl synthesis via direct arylation: Establishment of an efficient catalyst for intramolecular processes. *J. Am. Chem. Soc.* **2004,** 126, (30), 9186-9187.
57. Keller, T.; Gahlmann, T.; Riedl, T.; Scherf, U., Direct Arylation Polycondensation (DAP) Synthesis of Alternating Quaterthiophene-Benzothiadiazole Copolymers for Organic Solar Cell Applications. *Chempluschem* **2019,** 84, (9), 1249-1252.
